# Successful Management of Coronary Guidewire Fracture Using Intravascular Ultrasound—Guided Stent Jailing Technique During Retrograde Chronic Total Occlusion Recanalization

**DOI:** 10.1002/ccr3.71167

**Published:** 2026-02-02

**Authors:** Ang Gao, Guang‐Sheng Cai, Ji‐Hong Zou, Meng‐Yun Xu, Feng Qi, Xiao‐Juan Pan, Hong Qiu

**Affiliations:** ^1^ Department of Cardio‐Metabolic Medicine Center, Fuwai Hospital National Center for Cardiovascular Diseases, Chinese Academy of Medical Sciences and Peking Union Medical College Beijing China; ^2^ Department of Cardiology, Fuwai Yunnan Hospital, Chinese Academy of Medical Sciences Affiliated Cardiovascular Hospital of Kunming Medical University Kunming China

**Keywords:** coronary chronic total occlusion, guidewire fracture, intravascular ultrasound, percutaneous coronary intervention, stent jailing technique

## Abstract

Retrograde recanalization of chronic total occlusion can be challenging for its association with a greater risk of device entrapment and fracture. Aggressive advancement of retrograde guidewire through tortuous collateral may increase the risk of guidewire fracture. Stent jailing technique can sometimes be adopted based on the location, length, and morphology of the fractured guidewire when the patient was clinically stable and percutaneous attempts failed to retrieve. Intravascular ultrasound plays a critical role in assessing the length of the retained guidewire and guiding the final stenting to jail the wire filament into the vessel wall.

**Abbreviations**
CTA: computed tomography angiography
CTO: chronic total occlusion
EBU: Extra Back Up
IVUS: intravascular ultrasound
LAD: left anterior descending
LCX: left circumflex
PCI: percutaneous coronary intervention
PROGRESS CTO: Prospective Global Registry for the Study of Chronic Total Occlusion Intervention (PROGRESS CTO)
RCA: right coronary artery
SAL: Short Amplatz Left
UB3: Ultimate Bro 3

## 1 Introduction

Retrograde recanalization of coronary chronic total occlusion (CTO) can be challenging due to its association with a greater risk of device entrapment and fracture. Aggressive advancement of the retrograde guidewire through tortuous, angulated, or narrow collateral channels may increase the risk of wire entrapment and fracture. In addition, inappropriate manipulations, such as over‐rotation and forceful traction of the guidewire, were also predisposing factors of this complication [1]. In this report, we describe a case of guidewire fracture in the microcatheter following an unsuccessful attempt at CTO retrograde recanalization via the septal collateral branch, explore the potential causes, and discuss the feasibility of the stent jailing technique guided by intravascular imaging to manage this complication. Written consent had been acquired from the patient.

## 2 Case History and Examination

**FIGURE 1:**
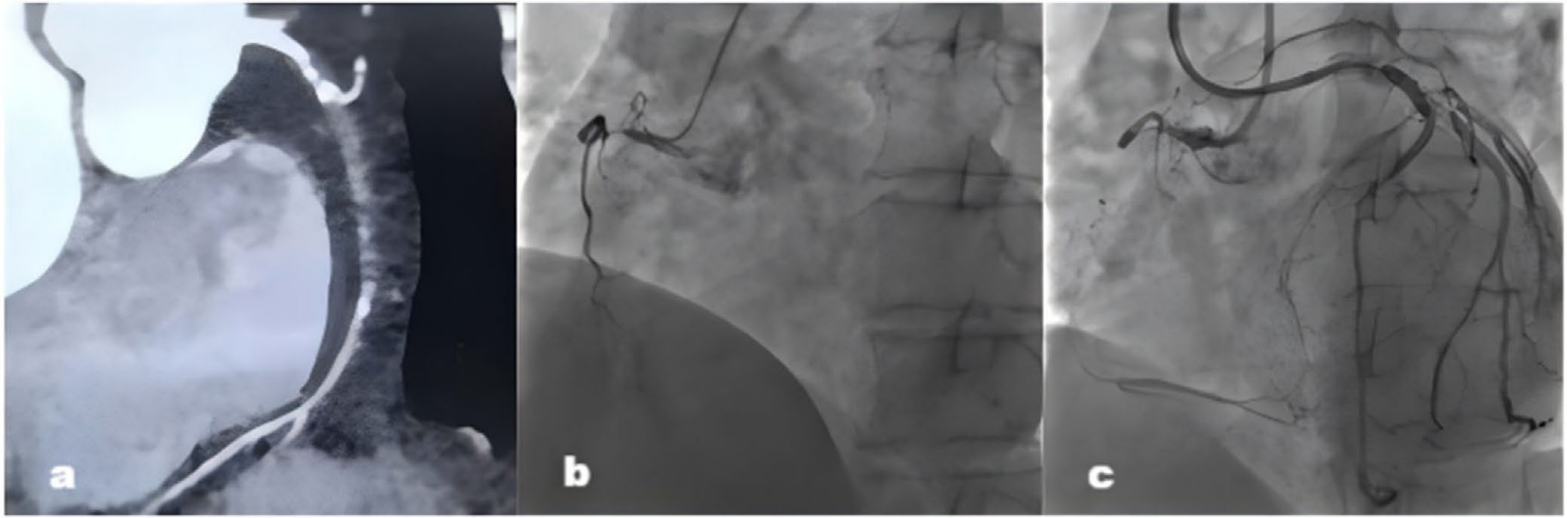
Coronary computed tomography angiography revealed calcification at the entry of occlusion and the length of the occlusion > 20 mm (a). Coronary angiography showed complete occlusion of the right coronary artery with a blunt entry, bending > 45° (b). The left anterior descending artery and left circumflex artery supplied tortuous and poorly developed collateral branches to the right coronary artery (c).

A 65‐year‐old man came to our hospital with a 2‐year history of chest distress. He had a medical history of stent implantation in the left anterior descending (LAD) and the left circumflex (LCX) artery. Right coronary artery (RCA) CTO recanalization was attempted because of progressive chest distress in the past 3 months. The 12‐lead electrocardiogram showed normal sinus rhythm with T wave inversion in the I, aVL, and V1–V4 leads. The echocardiogram revealed reduced left ventricular systolic function with an ejection fraction of 40% and a regional wall motion abnormality of the posterior septal wall, apical, and left ventricular anterior wall. Cardiac enzyme levels were within the normal range. The coronary computed tomography angiography (CTA) revealed calcification at the entry of occlusion and the length of the occlusion > 20 mm (Figure 1a). Coronary angiography showed complete occlusion of RCA with blunt entry, bending > 45° (Figure 1b). The LAD and LCX supplied tortuous and poorly developed collateral branches to the RCA (Figure 1c). The J‐CTO score was 4 points (calcification, blunt entry, bending > 45°, and occlusion length > 20 mm).

## 3 Treatment

**FIGURE 2:**
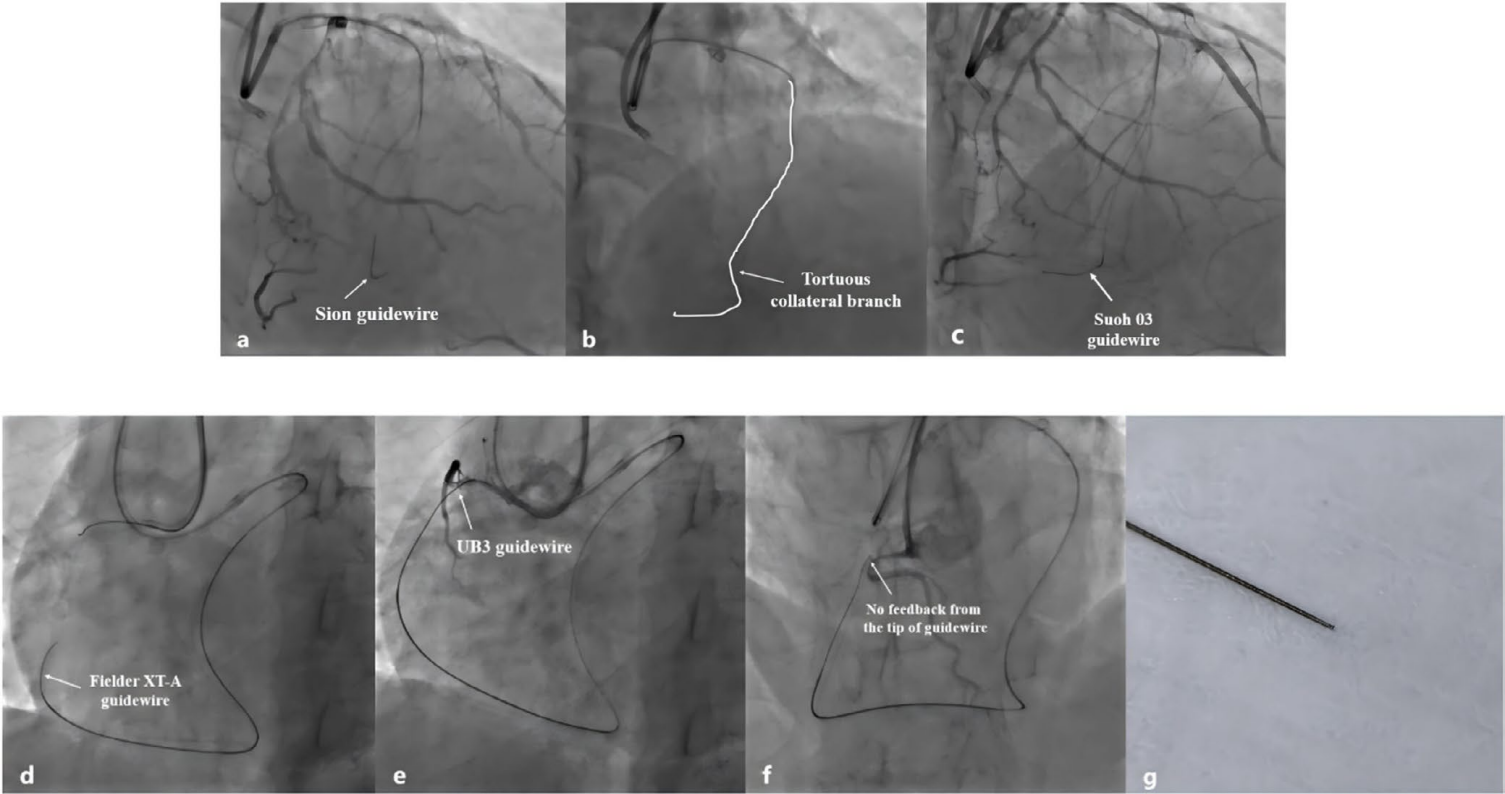
The Sion guidewire could not be advanced through the collateral branch to the distal RCA (a). The selective injection revealed extreme tortuosity of the septal collateral (b). The Suoh 03 guidewire passed through the tortuous channel with an acute angle (c). The Fielder XT‐A guidewire could not be advanced through the occlusion under the support of the microcatheter (d). The UB3 guidewire successfully reached the proximal RCA through the collateral branch (e). During the intervention, the UB3 guidewire might have fractured in the microcatheter because we found no feedback from the tip when we tried to rotate to adjust its direction (f). The tip of the retrieved retrograde guidewire was clean without the sign of unraveling (g).

**FIGURE 3:**
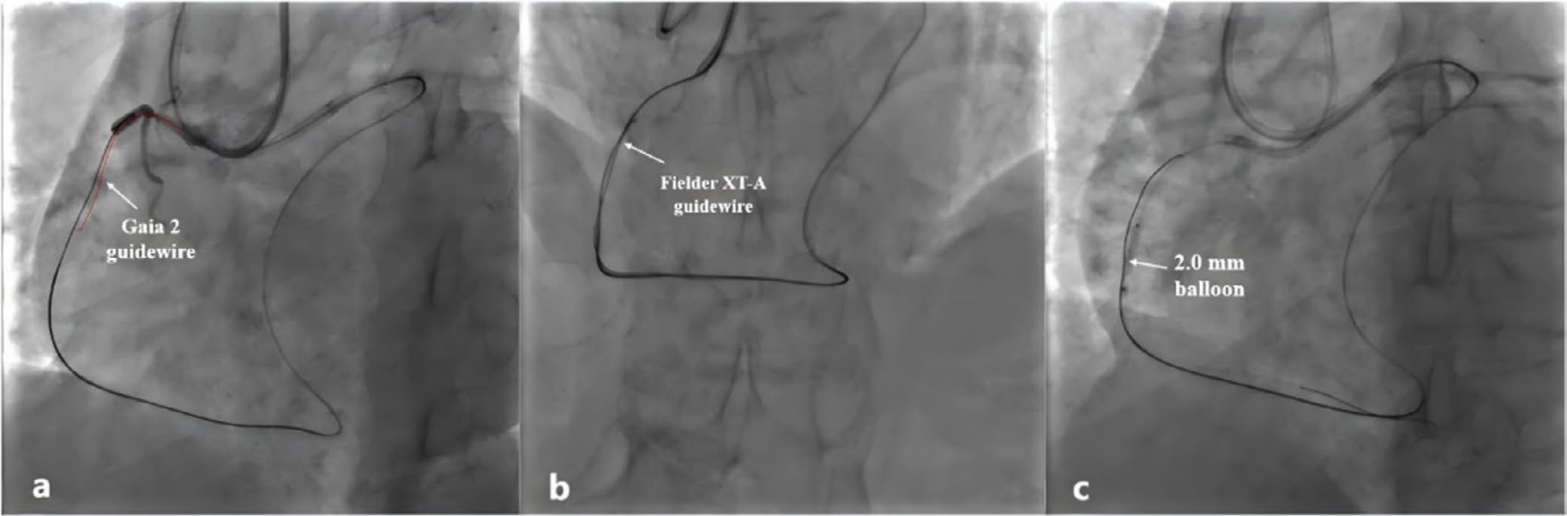
The antegrade Gaia 2 guidewire passed through the occlusion, but it failed to rendezvous with the retrograde guidewire (a). The Fielder XT‐A guidewire was again attempted to pass through the occlusion and rendezvoused well with the retrograde guidewire (b). A 2.0 mm balloon was progressively delivered and dilated from the distal to the proximal of the occlusion (c).

Given the patient's high J‐CTO score (4 points), we opted for an initial retrograde approach to recanalize the CTO lesion. The approach sites were both the radial arteries. We selected the 6F Short Amplatz Left (SAL) 1.0 (Medtronic Intecc) for the antegrade‐guiding catheter and the 7F Extra Back Up (EBU) 3.5 (Medtronic Intecc) for the retrograde‐guiding catheter. In view of the suboptimal retrograde channel, a Corsair Pro microcatheter (Asahi Intecc) was positioned at the proximal portion of the collateral channel to serve as a channel dilator and provide exceptional channel tracking as well as retrograde guidewire control. We initially selected the polymer‐jacketed Sion guidewire (Asahi Intecc) for retrograde crossing. However, the guidewire failed to be advanced through the collateral branch to the distal RCA (Figure 2a). We reassessed the distal path through selective injections from the microcatheter after several unsuccessful attempts at collateral channel surfing. The selective injections revealed extreme tortuosity of the septal collateral (Figure 2b), necessitating escalating the guidewire to a softer guidewire for improved trackability through the angulated segment. Finally, a Suoh 03 guidewire (Asahi Intecc) passed through the tortuous channel with an acute angle (Figure 2c). The microcatheter was then advanced and the guidewire was exchanged for a tapered tip Fielder XT‐A guidewire (Asahi Intecc) for transversing the occlusion, but it failed to pierce through the occlusion (Figure 2d). It was decided to escalate the guidewire to a non‐polymer coated intermediate tip‐load Ultimate Bro 3 (UB3) guidewire (Asahi Intecc) to enable active control, penetration power, and guidance within the occlusion, which can be advanced through the occlusion into the proximal RCA under the support of the microcatheter (Figure 2e). However, no tactile feedback was detected from the tip when we attempted to withdraw and rotate the wire to adjust its direction toward the antegrade wire (Figure 2f). We tried to retract the guidewire but found it had fractured in the microcatheter. The tip of the retrieved guidewire was clean without the sign of unraveling (Figure 2g). At this point, the antegrade approach was adopted to recanalize the occlusion. The Fielder XT‐A guidewire was initially attempted, but it failed to pass potentially because of the blunt entry stump. We then escalated the guidewire to the Gaia II to enhance penetration power. After several attempts, the wire finally pierced through the occlusion, but it failed to rendezvous with the retrograde guidewire (Figure 3a). The Fielder XT‐A guidewire with higher lubricity and trackability was again attempted to pass through the occlusion under the retrograde guidewire guidance (Figure 3b). A 2.0 mm balloon was progressively delivered and dilated from the distal to the proximal of the occlusion (Figure 3c).

**FIGURE 4:**
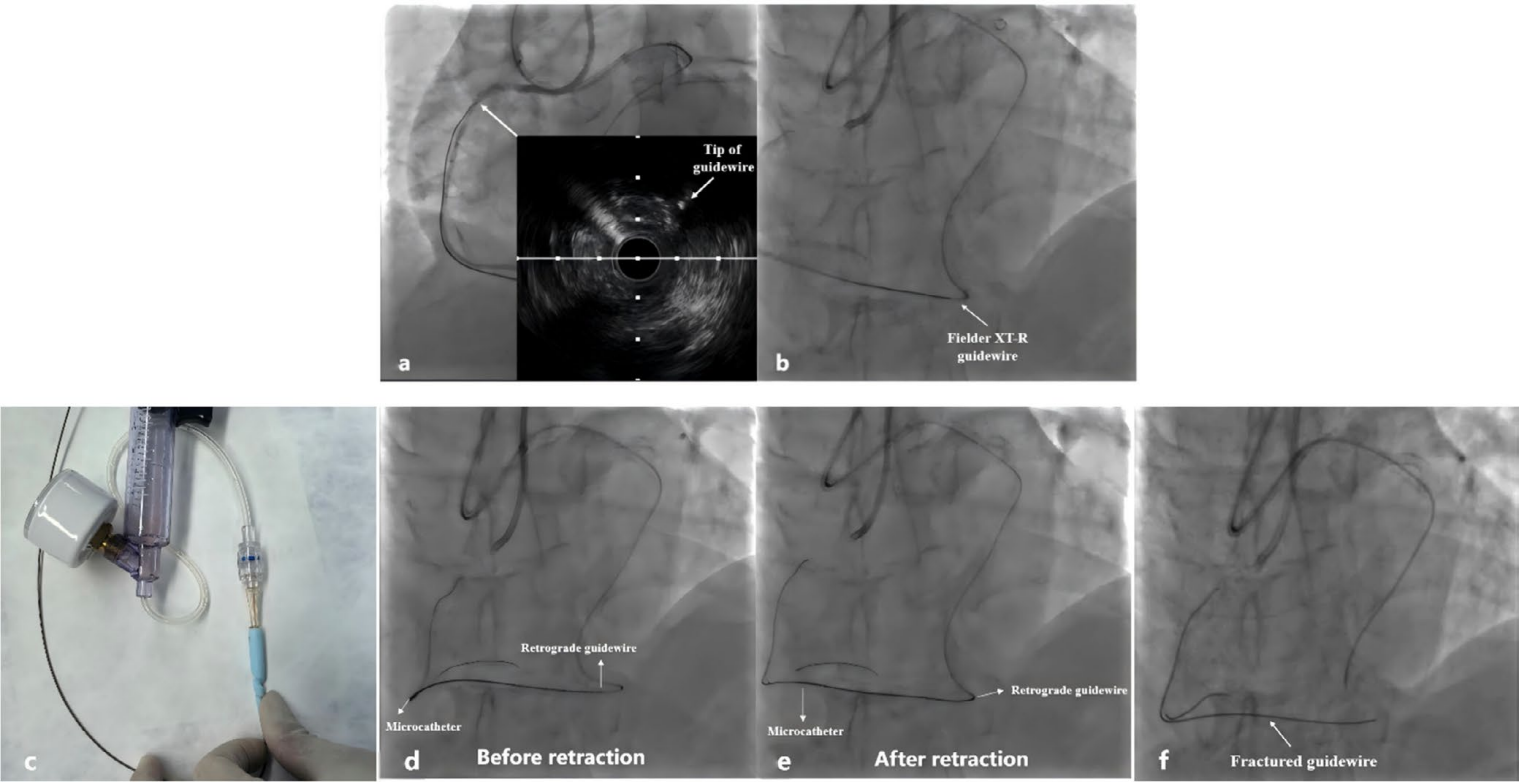
IVUS was performed to locate the exact position and assess the tip of the fractured remnant (a). Another Fielder XT‐R guidewire was advanced within the retrograde microcatheter to push the fractured remnant into the true lumen for aortic sinus snaring (b). The microcatheter was retracted with negative pressure (c). The fractured guidewire can only be partially retracted during microcatheter withdrawal (d: before retraction, e: after retraction). The guidewire remnant was left in situ (f).

We attempted the following methods to retrieve the fractured guidewire. First, intravascular ultrasound (IVUS) was performed to locate the exact position and assess the tip of the fractured remnant (Figure 4a). IVUS confirmed the tip of the UB3 guidewire was entrapped within the proximal calcified plaque but not in the false lumen, suggesting the potential for plaque traversal to re‐enter the true lumen. Accordingly, we tried to advance the Fielder XT‐R guidewire (Asahi Intecc) within the retrograde microcatheter to push the fractured remnant into the true lumen for aortic sinus snaring, but it failed because of the high resistance (Figure 4b). In addition, we also tried to retract the microcatheter with negative pressure (Figure 4c) to enhance the withdrawal force, but the fractured guidewire can only be partially retracted (Figure 4d,e). Finally, in view of the patient's stable clinical symptoms and the feasibility of conservative management of fractured remnants reported by previous cases, we decided to withdraw the retrograde microcatheter, leaving the fractured guidewire in situ (Figure 4f). The remnant was fixed to the vessel wall by the overlapping stents implantation (2.25 × 32 mm and 2.5 × 33 mm).

## 4 Outcome and Post‐Operative Follow‐Up

**FIGURE 5:**
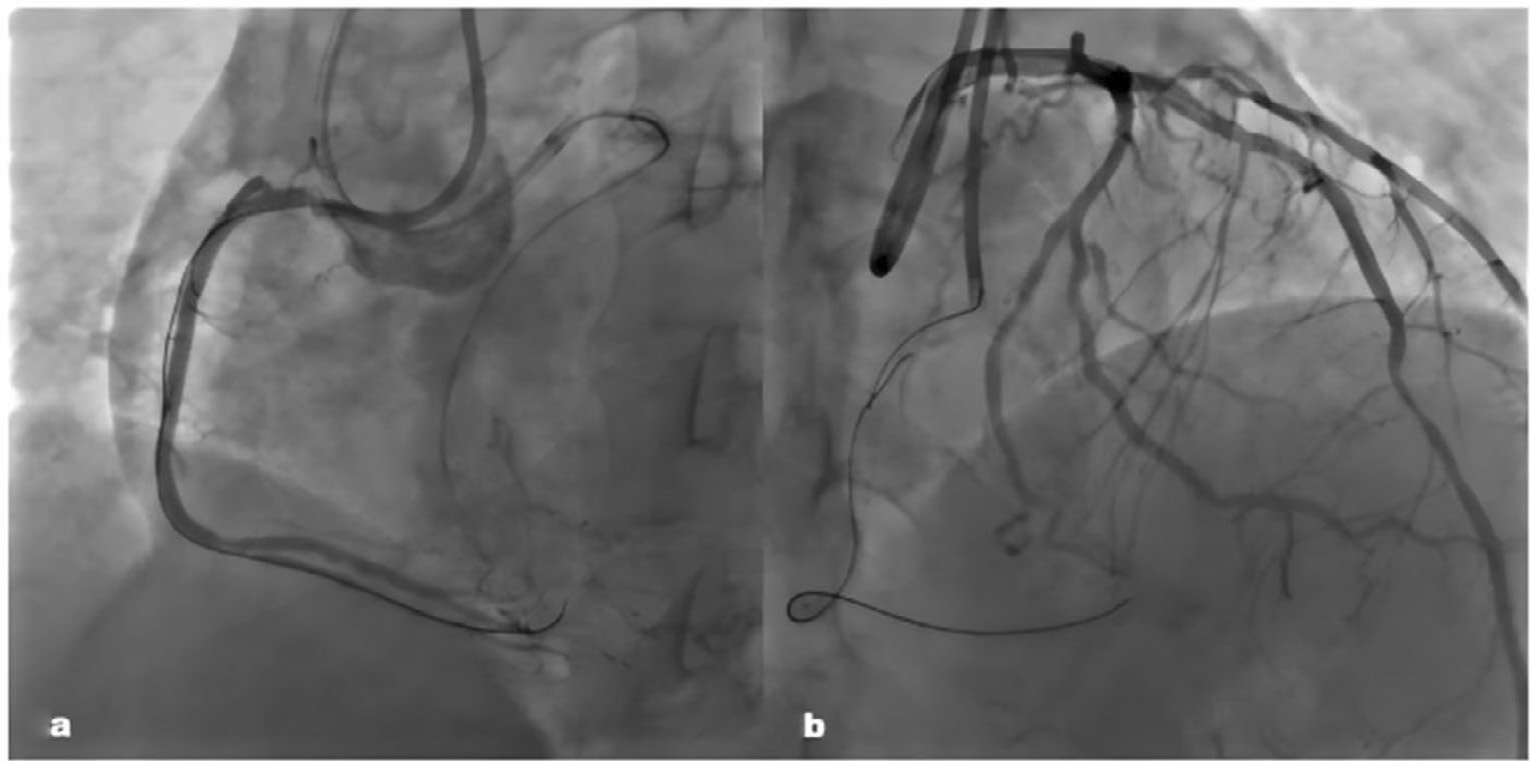
Post procedure angiographic images of the RCA (a). No damage to collateral branch was observed (b).

The final angiographic results were good and no damage to the collateral channel was observed (Figure 5). Repeated echocardiograms demonstrated no sign of pericardial effusion. The patient remained on dual antiplatelet therapy with aspirin and ticagrelor after discharge. He has remained asymptomatic for angina after discharge. At 6‐month follow‐up, dual antiplatelet therapy was discontinued and switched to clopidogrel monotherapy due to the occurrence of gastrointestinal bleeding.

## 5 Discussion

Although not frequently encountered during CTO percutaneous coronary interventions (PCIs), device entrapment/fracture was a potentially life‐threatening complication associated with thrombogenic and vascular injury risk. The reported rate of device entrapment/fracture during recanalization varied across different tertiary centers. Gasparini et al. reported a 1.5% incidence of device entrapment/loss in a study of 2361 CTO PCIs performed by five high‐volume CTO operators, with guidewires being the most commonly entrapment/fracture equipment (0.5%) [1]. Of 10,719 CTO PCIs in the Prospective Global Registry for the Study of Chronic Total Occlusion Intervention (PROGRESS CTO) registry, 0.2% of cases were complicated by guidewire entrapment/fracture [2]. Retrograde CTO PCIs might increase the risk of guidewire entrapment/fracture due to the presence of severely calcified, tortuous lesions or angulated collateral branches. The EuroCTO club documented a 0.6% rate of guidewire entrapment/fracture in 175 patients treated with the retrograde approach [3]. In a meta‐analysis comprising 3482 retrograde CTO PCIs from 26 studies, guidewire entrapment/fracture occurred in 1.2% of the cases [4]. In the PROGRESS CTO registry, comparative analysis demonstrated a significantly higher incidence of device entrapment/fracture in retrograde (65.0%) versus antegrade (29.0%) CTO PCIs (*p* < 0.001) while no difference in the rates of device entrapment between the two approaches was also reported [1, 2]. In addition, the incidence of guidewire entrapment/fracture might also be modulated by guidewire structural properties, particularly core material and polymer coating. Gasparini et al. reported that most guidewire entrapment/fracture occurred in stiffer and non‐polymer jacketed intermediate tip‐load wires, such as Gaia 2, Gaia 3, and UB3 guidewire [1]. In our case, the UB3 guidewire fracture occurred within the microcatheter during adjustment of the guidewire tip orientation. A diminutive, severely tortuous coronary collateral branch, multiple attempts to deliver the guidewire through the occlusion with an acute angle, and the rotational maneuvers collectively led to this complication. Acute angulation points in tortuous CC subject the guidewire to localized yield stress, thereby increasing fracture risk during delivery. Operator‐dependent factors during wire manipulation might also amplify intrinsic fracture risk during retrograde CTO PCI. Multiple attempts to advance and rotate the guidewire within the microcatheter to cross the occlusion may induce structural compromise, which potentiates fracture susceptibility. Strategies to avoid this complication include avoiding guidewire over‐rotation and forceful advancement. It's advisable to limit turns to alternating clockwise and counter‐clockwise rotation and avoid forceful retraction to free the entrapped guidewire [5].

To the best of our knowledge, this is the first reported case of guidewire fracture and retention in the microcatheter during retrograde CTO recanalization. The bailout stenting decision to jail the fragment in situ, on the basis of IVUS guidance and the patient's lack of symptoms, proved to be a safe and effective approach for non‐retrievable guidewire remnants. We summarized several operators' experiences for managing guidewire fracture during retrograde recanalization (Table 1). This case highlighted the pivotal role of clinical decision‐making flexibility, operator experience, and intravascular imaging in managing complications arising from CTO recanalization.

**TABLE 1:** Operator experience for managing guidewire fracture during retrograde CTO recanalization.

Prevention Guidewire should be cautiously manipulated through the collateral channel with an acute angle Over‐rotation and forceful traction should be avoided when the guidewire was entrapped within the occlusion
Management Intravascular imaging is pivotal for managing retained guidewire Complete retrieval of the fractured remnant should be ideally achieved Microcatheter can be retracted with negative pressure to increase the withdrawal force Prolonged attempts were not recommended if several percutaneous retrievals failed for the reason that extensive manipulation can lead to catastrophic coronary artery dissection and perforation Bailout stent jailing technique under intravascular imaging guidance can sometimes be adopted as an alternative approach for non‐retrievable guidewire remnant Emergent surgical should be considered if area at risk was large and the blood flow was compromised after failure of multiple interventional retrievals
Post‐operative The selection of antithrombotic strategy for retained guidewire depends on operator's evaluation of specific features such as fragment length and position, thrombotic risk and patients' comorbidities Dynamic assessment of bleeding risk and ischemic risk throughout long‐term follow‐up is necessary

Retained device fragment was a potentially life‐threatening complication since the remnants may provide a nidus for vascular endothelial injury and platelet deposition, leading to thrombosis formation, myocardial infarction, perforation, and pericardial tamponade [6, 7, 8, 9, 10]. Hence, complete retrieval of the fractured remnants should ideally be achieved. We summarized several cases with guidewire fracture during CTO recanalization in Table 2. The management of device entrapment/fracture in most cases can be attempted via interventional retrieval, such as snares, guidewire twirling, or biopsy forceps [8, 11, 12, 13], but the overall success rate is low. Data from the PROGRESS CTO registry reported retrieval was attempted in 71.4% of the entrapped cases, while the rate of complete retrieval was only 26.7% [2]. Conservative management strategies can sometimes be adopted depending on the location, length, and morphology of the fragment [14]. In our case, percutaneous retrieval of the fractured guidewire was initially adopted, but both snaring and wire twirling technologies were unsuccessful, potentially because of the calcified lesions. Given the patient was asymptomatic and hemodynamically stable, surgical retrieval was therefore ruled out. However, emergent cardiac surgery should be considered if the area at risk was large and the blood flow was compromised after the failure of multiple interventional modalities [15]. Additionally, perforation and cardiac tamponade caused by the fractured remnants, coronary rupture during attempted fragment removal, metal “bird's nest” created by the entanglement of the fragment with the implanted stent, and excessively long fragments protruding into the aorta were all indications for surgical intervention [5, 6, 7, 9]. Prolonged attempts at percutaneous retrieval were also not recommended for the reason that extensive manipulation can lead to potentially catastrophic coronary dissection and perforation [9, 11]. Paul previously reported a similar case in which the operator attempted to retrieve the fractured guidewire by using a triple wire technique after multiple snaring maneuvers failed. Although successful extraction of the fragment was ultimately achieved, the procedure induced coronary dissection and perforation [11]. Hence, leaving the wire fragment in situ could sometimes be adopted as an alternative approach. Although the thrombotic and perforation events were previously reported by several conservative treatment cases [7, 8], our case presented distinct clinical features. Kim et al. described a case in which uncoiled filaments were identified on IVUS. Despite the triple antiplatelet strategy and stent coverage, subacute thrombosis still occurred on the fourth day after discharge [8]. The surface of the fractured guidewire in the current case was clean without the sign of wire unraveling (unfolding of the tip coil into small filaments). IVUS plays a critical role in the conservative management strategy by assessing the length of the retained guidewire and guiding the final stenting to jail the wire filament into the vessel wall. We further performed IVUS to locate the exact position of the fractured wire and confirmed no wire coil unraveling occurred. Park et al. reported a case of pericardial tamponade caused by delayed penetration of a broken guidewire into the pericardium [7]. The remnant fragment in our case was not very long, and the tip was tightly jailed against the vessel wall through stent implantation under IVUS guidance, thereby mitigating the risk of wire migration into the pericardium.

**TABLE 2:** Cases with guidewire fracture during CTO recanalization.

Cases	Year	Age	Target vessel	Crossing strategies	Guidewire	Causes of fracture	Retrieval technique	Complete retrieval	Complications	Follow‐up
Sianos et al.	2010	68	LAD	Retrograde	Fielder	Aggressive removal the entrapped guidewire	Balloon inflation, guidewire twirling	No	None	Asymptomatic during 12‐month follow‐up
Danek et al.	2015	69	LAD	Antegrade	Fielder XT	Aggressive removal the entrapped guidewire	Advancement of microcatheter over the entrapped guidewire	Yes	None	Not available
Park et al.	2015	55	RCA	Retrograde	Fielder XT‐R	Excessive rotation, tortuous collateral channel	Not attempted	No	Pericardial tamponade	Surgical removal of the guidewire
Baumann et al.	2017	61	RCA	Retrograde	Gaia 3	Aggressive pulling the entrapped guidewire	Balloon inflation, snaring	No	No	Asymptomatic during 6‐month follow‐up
Cho et al.	2018	75	RCA	Antegrade	Gaia 3	Use of atherectomy to free the entrapped wire	Forceful pulling, snaring, rotational atherectomy	No	No	Not available
Li et al.	2022	51	RCA	Not available	Gaia 2	Not available	Snaring, rotational atherectomy	No	No	Stable during 3‐month follow‐up
Li et al.	2022	75	RCA	Not available	Sion	Jailing of guidewire during stenting	Snaring, rotational atherectomy	No	No	Stable during 7‐month follow‐up

In our case, the patient accepted dual antiplatelet therapy with aspirin and ticagrelor. During follow‐up, no coronary events were observed; however, he experienced gastrointestinal bleeding. Currently, there are no standards in regards to the choice of antithrombotic strategies for retained guidewire in the coronary artery. The selection of antithrombotic strategy for retained guidewire in previous cases primarily depends on the operator's comprehensive evaluation of specific features such as fragment length and position, thrombotic risk, and patients' comorbidities. Dual antiplatelet therapy seemed to be sufficient if the fragment was not very long and retained within the distal or the small branch of the artery in most cases [14, 16, 17]. Triple antithrombotic strategies (dual antiplatelet + cilostazol or warfarin) were also reported in patients with high‐risk clinical features such as long wire remnants, wire fragments protruding into the aorta, and uncoiled filaments [8, 18]. Li et al. proposed that prolonged dual antiplatelet therapy (usually aspirin + ticagrelor) seemed to be safe and effective based on the summary of three entrapped cases [16]. The patient in our case presented with multivessel disease and a previous history of myocardial infarction. Given the absence of high‐risk bleeding factors, we opted for dual antiplatelet therapy with aspirin plus ticagrelor after stent implantation. Although unexpected gastrointestinal bleeding occurred during the follow‐up, it's better to initiate potent antiplatelet therapy, especially now with a fractured guidewire retained in the artery. In addition, the occurrence of gastrointestinal bleeding also stressed the necessity for long‐term follow‐up. Most cases on retained intracoronary guidewire highlighted the necessity for intensified and prolonged antithrombotic therapy, which may increase the patient's bleeding risk [8, 16, 18]. Hence, a tailored antithrombotic strategy based on a dynamic assessment of bleeding and ischemic risk throughout the follow‐up period was clinically imperative.

## 6 Conclusion

According to this case, we concluded that although percutaneous retrieval was often the first choice to manage the fractured guidewire during CTO PCI, the IVUS‐guided stent jailing technique can sometimes be an alternative approach if the patient is clinically stable and the remnant is not very long and poses no significant risk to the coronary flow. Antithrombotic management for the retained guidewire should be individualized depending on the comprehensive evaluation of bleeding and thrombotic risks.

## Author Contributions

**Ang Gao:** conceptualization, data curation, formal analysis, software, writing – original draft. **Guang‐Sheng Cai:** data curation, formal analysis, project administration, resources, software. **Ji‐Hong Zou:** investigation, project administration, visualization. **Meng‐Yun Xu:** data curation, writing – review and editing. **Feng Qi:** supervision, writing – review and editing. **Xiao‐Juan Pan:** formal analysis, funding acquisition, investigation, supervision, visualization, writing – review and editing. **Hong Qiu:** conceptualization, formal analysis, project administration, resources, supervision, writing – review and editing.

## Ethics Statement

This study has been approved by the ethics committee of Yunnan Fuwai Hospital.

## Consent

We confirmed that oral and written consent had been acquired from the patient.

## Conflicts of Interest

The authors declare no conflicts of interest.

## Data Availability

The materials mentioned above are available from the authors upon reasonable request.
